# Viral aetiology of acute respiratory infections among children and associated meteorological factors in southern China

**DOI:** 10.1186/s12879-015-0863-6

**Published:** 2015-03-13

**Authors:** Binglin Cui, Dangui Zhang, Hui Pan, Fan Zhang, Jeremy Farrar, Frieda Law, H Rogier van Doorn, Beiyan Wu, William Ba-Thein

**Affiliations:** Pediatric Department, the First Affiliated Hospital of Shantou University Medical College, Shantou, Guangdong P.R. China; The Research Center of Translational Medicine, the Second Affiliated Hospital of Shantou University Medical College, Shantou, Guangdong P.R. China; Shantou-Oxford Clinical Research Unit, Shantou University Medical College, Shantou, Guangdong P.R. China; Oncology Research Laboratory, Cancer Hospital of Shantou University Medical College, Shantou, Guangdong P.R. China; Oxford University Clinical Research Unit, Hospital for Tropical Diseases, Wellcome Trust Major Overseas Programme, Ho Chi Minh City, Viet Nam; Consultant Office, Shantou University Medical College, Shantou, Guangdong P.R. China; Department of Microbiology and Immunology, Shantou University Medical College, Shantou, Guangdong 515041 P.R. China

**Keywords:** Virus, Acute respiratory infection, Meteorological factor, Children, China

## Abstract

**Background:**

Acute respiratory infections (ARIs) are common in children and mostly caused by viruses, but the significance of the detection of multiple viruses in ARIs is unclear. This study investigated 14 respiratory viruses in ARIs among children and associated meteorological factors in Shantou, southern China.

**Methods:**

Paired nasal/throat-flocked swabs collected from 1,074 children with ARIs, who visited outpatient walk-in clinics in a tertiary hospital between December 2010 and November 2011, were examined for fourteen respiratory viruses - influenza viruses (FluA, FluB), respiratory syncytial viruses (RSV A and B), human coronaviruses (hCoV: 229E, OC43, HKU1, NL63), human metapneumoviruses (hMPV A and B), parainfluenza viruses (PIV1-4), human rhinoviruses (HRV A, B, C), enteroviruses (EV), adenoviruses (ADV), human bocavirus (hBoV), and human parechoviruses (hPeV) - by multiplex real-time PCR.

**Results:**

We identified at least one virus in 82.3% (884/1,074) and multiple viruses in 38.6% (415/1,074) of patients. EV and HRV were the most frequently detected single viruses (42.3%, 374/884 and 39.9%, 353/884 respectively) and co-detected pair (23.1%, 96/415). Overlapping seasonal trends of viruses were recorded over the year, with dual peaks for EV and single peaks for the others. By logistic regression analysis, EV was positively associated with the average temperature and humidity, hCoV, and PIV4, but negatively with HRV, PIV3, and hBoV. HRV was inversely associated with EV and PIV3.

**Conclusions:**

This study reports high viral detection and co-detection rates in pediatric ARI cases mainly due to EV and HRV. Many viruses circulated throughout the year with similar seasonal trends in association with temperature, humidity, and wind velocity. Statistically significant associations were present among the viruses. Understanding the polyviral etiology and viral interactions in the cases with multiple viruses warrants further studies.

**Electronic supplementary material:**

The online version of this article (doi:10.1186/s12879-015-0863-6) contains supplementary material, which is available to authorized users.

## Background

Acute respiratory infections (ARIs) are one of the illnesses of highest morbidity and mortality in children worldwide [1-3]. The pathogens causing ARIs vary geographically and by season, but globally viruses play a major role. Respiratory syncytial virus (RSV) is by far the most common pathogen associated with severe respiratory diseases as bronchiolitis, exacerbation of asthma, or pneumonia in early life, and is a leading cause of hospitalization in children under two [4]. Influenza viruses have the greatest potential to cause severe respiratory diseases in the very young, the elderly and those with underlying chronic conditions [5]. Enteroviruses including human rhinoviruses (HRV) and human enteroviruses (EV), previously identified in childhood upper respiratory tract infections, are commonly associated with milder ARIs and have been suspected as major etiological agents of lower respiratory tract infections leading to bronchiolitis and pneumonia in infants [6]. It has also been reported that human metapneumovirus (hMPV) causes approximately 5-10% of all ARIs in children and adults [7] and adenoviruses (ADV) account for 5-15% of respiratory infections in children [8]. Respiratory illnesses can be attributable to other viruses such as parainfluenza viruses (PIV) and human coronaviruses hCoV-229E, OC43 [7]. With rapid progress in molecular diagnostics, newly discovered viruses including human bocavirus (hBoV), human coronaviruses (hCoV-NL63, hCoV-HKU1), human parechoviruses (hPeV), and polyomaviruses WU (WUPyV) and KI (KIPyV) have also been detected in children with respiratory infections, with varying levels of proof of causation [9].

Hospital-based studies in children published over the last decade worldwide have identified viruses in up to 95% of ARI episodes, with a single virus found in 40-60% and multiple viruses in 1-40% of infected patients [7,8,10]. Co-infection is reportedly related to the time of year when circulations of multiple viruses occur [11]. Some studies have shown that the prevalence of co-infections is not related to the absolute prevalence of individual viruses [12]. Factors such as young age, male gender, and history of immunosuppression are associated with an increased chance of viral co-infections [11,13,14]. There could be likely interactions between climatic, environmental, and behavioral factors, and complex interplay between circulating viruses and population-level immunity regarding viral co-infections. Understanding these factors may help us prevent transmission of these infections.

Recent etiologic studies on pediatric respiratory infections mostly report the prevalence in hospitalized children and the seasonality of viruses without elaborating viral co-infection. Therefore, the significance of the detection of multiple viral pathogens in ARIs is unclear. Here, we investigated fourteen common respiratory viruses among pediatric outpatients in southern China during 2010–2011 and their associations with meteorological factors.

## Methods

### Study location

This study was conducted at the Pediatric Outpatient Walk-in Clinics, the First Affiliated Hospital of Shantou University Medical College. The Pediatric Department provides both primary and tertiary care (common practice in China) for approximately 35,000 children per year in the Chaoshan region of southern China. The Chaoshan region is in the subtropical zone with an average annual temperature of 21.3°C and excellent to lightly polluted air quality levels (air quality index, AQI: 17–142, in 2012–2013).

### Study design

Based on modified WHO standard case definition of ARIs [7], eligible participants were defined as a child 0–16 years of age presenting within 3 days of onset of illness with at least two of the following: fever, sore throat, cough, rhinorrhea, nasal congestion, and hoarseness of voice. Patients with any condition preventing swab collection were excluded. We recruited eligible patients in the morning, during which approximately 70% of patient visits are made, on a daily basis except public holidays from December 2010 to November 2011. Participants’ demographic details and clinical features are shown in Table 1. Paired nose and throat-flocked swabs (Copan, Brescia, Italy, Cat. no. 503CS01 and 502CS01) were collected from each participant, combined in one tube, and stored within 3 h of collection at −80°C until further processing.

**Table 1:** **Demographic and clinical characteristics, and virus positivity of pediatric outpatients with acute respiratory infections (n = 1,074)**

**Parameter**	**Patients with an ARI**	**Virus-negative cases**	**Virus-positive cases**					**OR (95% CI)**	***p value*** ^**f**^
	**Total (n = 1,074)**	**Total (n = 190)**	**Total (n = 884)**	**OR (95% CI)**	***p value*** ^**e**^	**Single virus (n = 469)**	**Multiple viruses (n = 415)**		
**Gender**									
Male	669 (62.3)	107 (56.3)	562 (63.6)	Reference	-	293 (62.5)	269 (64.8)	Reference	-
Female	405 (37.7)	83 (43.7)	322 (36.4)	0.7 (0.5-1.0)	0.06	176 (37.5)	146 (35.2)	0.9 (0.7-1.2)	0.47
**Age group (year)**									
≤1 (infant)	225 (20.9)	41 (21.6)	184 (20.8)	Reference	-	103 (22.0)	81 (19.5)	Reference	-
>1 -2 (toddler)	201 (18.7)	31 (16.3)	170 (19.2)	1.2 (0.7-2.0)	0.44	86 (18.3)	84 (20.2)	1.2 (0.8-1.9)	0.31
>2-5 (preschool child)	468 (43.6)	86 (45.3)	382 (43.2)	1.0 (0.7-1.5)	1.00	206 (43.9)	176 (42.4)	1.1 (0.8-1.5)	0.65
>5-16 (school child)	180 (16.8)	32 (16.8)	148 (16.7)	1.0 (0.6-1.7)	0.92	74 (15.8)	74 (17.8)	1.3 (0.8-2.0)	0.28
**Clinical presentation** ^a^									
Cough	894 (83.2)	161 (84.7)	733 (82.9)	0.9 (0.6-1.3)	0.54	384 (81.9)	349 (84.1)	1.2 (0.8-1.7)	0.38
Rhinorrhea	666 (62.0)	117 (61.6)	549 (62.1)	1.0 (0.7-1.4)	0.89	273 (58.2)	276 (66.5)	1.4 (1.1-1.9)	<0.05
Fever of >38°C^b^	359 (33.4)	75 (39.5)	284 (32.1)	0.7 (0.5-1.0)	0.05	138 (29.4)	146 (35.2)	1.3 (1.0-1.7)	0.08
Nasal congestion	157 (14.6)	36 (18.9)	121 (13.7)	0.7 (0.5-1.0)	0.06	65 (13.9)	56 (13.5)	1.0 (0.7-1.4)	0.89
Sneezing	109 (10.1)	22 (11.6)	87 (9.8)	0.8 (0.5-1.4)	0.47	48 (10.2)	39 (9.4)	0.9 (0.6-1.4)	0.68
Sore throat	99 (9.2)	19 (10.0)	80 (9.0)	0.9 (0.5-1.5)	0.68	43 (9.2)	37 (8.9)	1.0 (0.6-1.5)	0.89
Others^c^	57 (5.3)	14 (7.4)	43 (4.9)	0.6 (0.3-1.2)	0.16	23 (4.9)	20 (4.8)	1.0 (0.5-1.8)	1.00
**Socio-medical information** ^a^									
ARI history in 4 weeks	472 (43.9)	90 (47.4)	382 (43.2)	0.8 (0.6-1.2)	0.29	193 (41.2)	189 (45.5)	1.2 (0.9-1.6)	0.19
Passive smoking	605 (56.3)	103 (54.2)	502 (56.8)	1.1 (0.8-1.5)	0.52	275 (58.6)	227 (54.7)	0.9 (0.7-1.1)	0.24
Family size (person, n = 1,072)^d^									
≤3	287 (26.8)	44 (23.3)	243 (27.5)	Reference	-	117 (25.0)	126 (30.4)	Reference	-
4-15	785 (73.2)	145 (76.7)	640 (72.5)	0.8 (0.6-1.2)	0.23	351 (75.0)	289 (69.6)	0.8 (0.6-1.0)	0.08

Data are presented as number (%). ARIs, acute respiratory infections.

^a^Absence of respective symptoms, ARI history, or exposure to passive smoking as reference.

^b^Armpit temperature.

^c^Including hoarseness of voice, headache, earache, conjunctivitis, oral ulcer, peri-oral blebs, vomiting, diarrhea, stomachache, and rash.

^d^Two missing data.

^e^Comparison between virus-negative and virus-positive groups.

^f^Comparison between single-virus and multiple-virus groups.

### Laboratory procedure

Multiplex real-time PCR was performed using Roche, Lightcycler 480 II (Roche Diagnostics, Penzberg, Germany) to identify the following 14 respiratory viruses: influenza A (FluA), influenza B (FluB), respiratory syncytial viruses A and B (RSV), human coronaviruses 229E, OC43, HKU1 and NL63 (hCoV), human metapneumoviruses A and B (hMPV), human parainfluenza virus types 1, 2 , 3, and 4 (PIV1, PIV2, PIV3, and PIV4), human rhinoviruses A, B, and C (HRV), human enteroviruses (EV), human adenoviruses (ADV), human bocavirus (hBoV), and human parechoviruses (hPeV).

Nucleic acid extraction was performed using the QIAamp Viral RNA Mini Kit (QIAGEN GmbH, Hilden, Germany, Cat. no. 52906). Reverse transcription and Real-time PCR assays were performed as described previously [15], except for the primers and/or probes for HRV, hPeV, and internal control equine arteritis virus (EAV, see the sequences of 14 viruses in Additional file 1). Due to known cross-reactivity between enteroviruses [16-18], HRV was detected using two sets of primers and probes: HRV-v1 (version 1) for screening and HRV-v2 (version 2) for confirmation. Real-time PCR results were interpreted as described previously [15]. The PCR was considered positive or negative when the Cp value was less than 40 cycles or exceeded 40 cycles, respectively, and the positive control showed the expected Cp value, negative control was negative, and internal control showed the expected Cp value. A negative internal control signal was accepted in case of a positive target sequence with correct positive and negative control signals.

### Meteorological data

Meteorological data, including the average daily temperature (°C), the average daily humidity (%), and the average daily wind velocity (km/h), were collected from the official website of Shantou Meteorology, TuTiempo.net (http://www.tutiempo.net/en/Climate/Shantou/2011/593160.htm).

### Statistical analysis

We used Chi-square test to compare differences in the distribution of categorical variables, ANOVA and Kruskall Wallis tests to compare medians, and the Pearson correlation analysis to evaluate the associations between the meteorological factors and viruses and among viruses. The variables with significant associations were further analyzed in multivariate logistic regression models, in which symptoms and positivity of viruses were treated as dependent and independent variables to assess virus-symptom associations; and individual viruses were treated as dependent variables with meteorological factors or other viruses as independent variables to investigate meteorological factor-virus and virus-virus associations. A two-tailed *p-Value* of <0.05 was considered significant. All these analyses were performed with SPSS Statistics version 17.0.

### Ethics

The study was approved by the Ethics Committee of the First Affiliated Hospital of Shantou University Medical College and the Oxford University Tropical Research Ethical Committee (OxTREC). Written informed consent was obtained from parents or legal guardians of children enrolled in the study.

## Results

Of 1,074 children (62.3% male) recruited, 43.6% (468/1,074) were >2-5 years old (Table 1). At least one virus was identified in 82.3% (884/1,074) of the patients, with single virus in 43.7% (469/1,074) and multiple viruses in 38.6% (415/1,074). hPeV was not detected. Compared with virus-negative patients, virus-positive patients were less likely to have fever (OR: 0.7, 95% CI: 0.5-1.0, *p* = 0.05). Patients with multiple viruses were more likely to have rhinorrhea than those with single virus (OR: 1.4, 95% CI: 1.1-1.9, *p* < 0.05, Table 1). hCoV (OR: 1.6, 95% CI: 1.0-2.4) and PIV4 (OR: 1.6, 95% CI: 1.0-2.4) were more prevalent in the >5 year age group than in the ≤5 year group (all *p* ≤ 0.05), while hBoV (OR: 0.3, 95% CI: 0.1-0.7) and RSV (OR: 0.4, 95% CI: 0.2-1.0) were less frequently found in the >5 year group (all *p* < 0.05). Chi-square test and multivariate logistic regression analysis showed that cough was positively associated with HRV and RSV, and negatively with EV; rhinorrhea was positively associated with HRV, PIV3, and hBoV, and negatively with EV; fever was positively associated with EV, and negatively with HRV and PIV3; and nasal congestion was positively associated with RSV, and negatively with EV and hCoV (all *p* < 0.05, Table 2).

**Table 2:** **Multivariate logistic regression analysis of associations between viruses and clinical presentations**

**Variable**	**Cough (n = 894)**		**Rhinorrhea (n = 666)**		**Fever (>38°C, n = 359)**		**Nasal congestion (n = 157)**	
	**n (%)**	**OR (95% CI)**	**n (%)**	**OR (95% CI)**	**n (%)**	**OR (95% CI)**	**n (%)**	**OR (95% CI)**
**EV**	271 (30.3)	0.4 (0.3-0.5)	178 (26.7)	0.5 (0.4-0.7)	153 (42.6)	1.4 (1.1-1.9)	30 (19.1)	0.4 (0.3-0.7)
**HRV**	313 (35.0)	1.7 (1.2-2.6)	242 (36.3)	1.6 (1.2-2.1)	80 (22.3)	0.5 (0.3-0.6)		
**hCoV**							12 (7.6)	0.5 (0.3-1.0)
**PIV3**			120 (18.0)	3.0 (1.8-4.7)	34 (9.5)	0.6 (0.4-0.9)		
**hBoV**			67 (10.1)	3.3 (1.7-6.5)				
**RSV**	72 (8.1)	4.3 (1.3-14.0)					20 (12.7)	2.0 (1.2-3.6)

Only the variables with p < 0.05 are shown (absence of non-virus cases as reference[s] accordingly).

*Abbreviations:* EV, enterovirus; HRV, human rhinovirus; hCoV, human coronavirus; PIV3, parainfluenza 3; hBoV, human bocavirus; RSV, respiratory syncytial virus.

Viruses detected alone or co-detected with other viruses are shown in Table 3. The most frequently detected virus was EV (42.3%, 374/884), followed by HRV (39.9%, 353/884), and hCoV (17.5%, 155/884). EV and HRV were most commonly co-detected with other viruses (Table 3) and also the most commonly co-detected pair of viruses (23.1%, 96/415, see the distribution pattern of viruses in Additional file 2). Screening with HRV-v1 identified 298 cases co-positive for HRV and EV, and subsequent confirmation with HRV-v2 primers/3 probes [15] resulted in only 96 positive cases (32.2%, 96/298).

**Table 3:** **Detection of viruses from nasal/throat swabs of pediatric outpatients with acute respiratory infections, ARIs (n = 884)**

**Positive for**	**Total (n = 884)**	**EV (n = 374)**	**HRV (n = 353)**	**hCoV (n = 155)**	**PIV3 (n = 145)**	**PIV4 (n = 132)**	**hBoV (n = 78)**	**RSV (n = 75)**	**FluA (n = 58)**	**FluB (n = 54)**	**PIV2 (n = 40)**	**PIV1 (n = 22)**	**ADV (n = 19)**	**hMPV (n = 3)**
**Single virus**	469 (53.1)	170 (36.2)	161 (34.3)	16 (3.4)	41 (8.7)	10 (2.1)	24 (5.1)	6 (1.3)	6 (1.3)	22 (4.7)	5 (1.1)	3 (0.6)	4 (0.9)	1 (0.2)
**Multiple viruses**	415 (46.9)	204 (49.2)	192 (46.3)	139 (33.5)	104 (25.1)	122 (29.4)	54 (13.0)	69 (16.6)	52 (12.5)	32 (7.7)	35 (8.4)	19 (4.6)	15 (3.6)	2 (0.5)
2 viruses	265 (30.0)	132	122	51	56	44	31	27	22	18	9	12	5	1
3 viruses	111 (12.6)	53	51	62	31	52	16	18	12	10	15	5	7	1
4 viruses	27 (3.1)	10	17	15	11	14	7	12	9	3	5	2	3	0
5 viruses	5 (0.6)	4	0	4	3	5	0	5	4	0	0	0	0	0
6 viruses	6 (0.7)	4	2	6	2	6	0	6	5	0	5	0	0	0
7 viruses	1 (0.1)	1	0	1	1	1	0	1	0	1	1	0	0	0

Data are presented as number (%).

*Abbreviations:* EV, enterovirus; HRV, human rhinovirus; hCoV, human coronavirus; PIV1-4, parainfluenza 1–4; hBoV, human bocavirus; RSV, respiratory syncytial virus; FluA, influenza A; FluB, influenza B; ADV, adenovirus; hMPV, human metapneumovirus.

### Seasonality and meteorological factors

**Figure 1:**
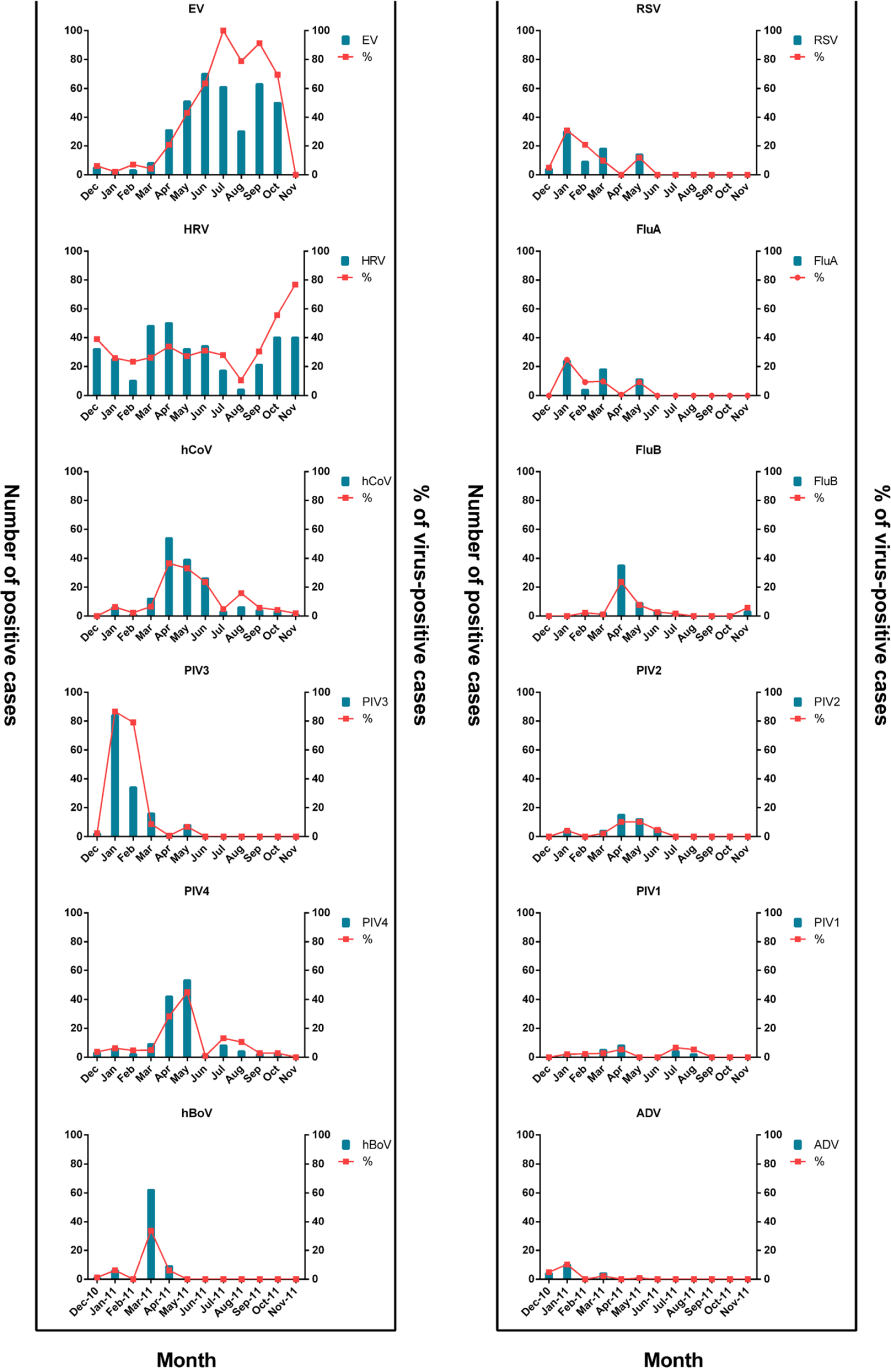
**Temporal circulation pattern of respiratory viruses (n = 1,074).** EV, enterovirus; HRV, human rhinovirus; hCoV, human coronavirus; PIV1-4, parainfluenza 1–4; hBoV, human bocavirus; RSV, respiratory syncytial virus; FluA, influenza A; FluB, influenza B; ADV, adenovirus. % = individual-virus-positive cases/total cases tested.

**Figure 2:**
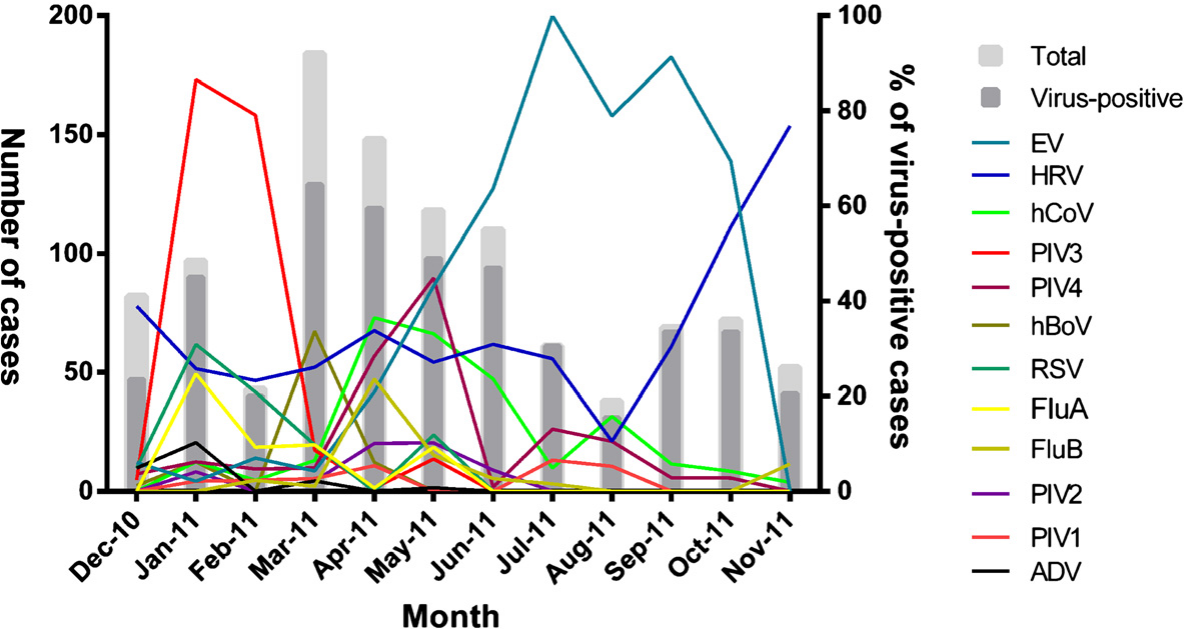
**Temporal co-circulation pattern of respiratory viruses (n = 1,074).** EV, enterovirus; HRV, human rhinovirus; hCoV, human coronavirus; PIV1-4, parainfluenza 1–4; hBoV, human bocavirus; RSV, respiratory syncytial virus; FluA, influenza A; FluB, influenza B; ADV, adenovirus. % = individual-virus-positive cases/total cases tested.

The temporal circulation and co-circulation patterns of viruses are shown in Figures 1 and 2. There were overlapping seasonal trends of many viruses throughout the year, with dual peaks for EV in July and September and single peaks for the other viruses. Both EV and HRV circulated throughout the year. hCoV and PIV4 circulated predominantly between April and May but sporadically throughout the year. PIV3, RSV, FluA, and ADV peaked in January, while hBoV peaked in March. FluB circulated mostly from February to July with a peak in April. Co-detection of 5–7 viruses occurred all in May (see Additional file 3). The optimal average daily temperature, humidity, and wind velocity for these viruses are shown in Table 4.

**Table 4:** **The optimal average daily temperature, humidity, and wind velocity for some circulating viruses**

**Virus**	**Average daily temperature (** **°C)**			**Average daily humidity (%)**			**Average daily wind velocity (Km/h)**	
	**5-14.9**	**15-24.9**	**25-35**	**30-49**	**50-69**	**70-99**	**3-12.9**	**13-22.9**
EV	**-**	**-**	**√**	**-**	**-**	**√**	**√**	**-**
HRV	**-**	**√**	**-**	**-**	**√**	**√**	**-**	**√**
hCoV	**-**	**√**	**√**					
PIV3	**√**	**-**	**-**		**√**	**√**	**√**	**-**
PIV4	**-**	**√**	**-**	**-**	**√**	**√**	**√**	**-**
hBoV	**-**	**√**	**-**	**√**	**√**	**-**		
RSV	**√**	**-**	**-**					
FluA	**√**	**-**	**-**					
FluB	**-**	**√**	**-**	**-**	**√**	**-**	**√**	**-**
PIV2				**-**	**-**	**√**		
ADV	**√**	**-**	**-**					

Only statistically significant results (p < 0.05 by Chi-square test for individual comparisons of proportions within each group) are shown as “**√**” with reference(s) shown as “-”. The optimal temperature for hCoV and hBoV was 15–35°C. The optimal relative humidity was 60-79% for PIV3 and 80-99% for hCoV.

*Abbreviations:* EV, enterovirus; HRV, human rhinovirus; hCoV, human coronavirus; PIV2-4, parainfluenza 2–4; hBoV, human bocavirus; RSV, respiratory syncytial virus; FluA, influenza A; FluB, influenza B; ADV, adenovirus.

### Virus-meteorological and virus-virus associations

Table 5 shows the multivariate logistic regression models for independent associations between the viruses and meteorological factors and between the viruses. EV was positively associated with the average temperature and humidity and the presence of hCoV and PIV4, but negatively with HRV, PIV3, and hBoV. HRV was negatively associated with the presence of EV and PIV3. hCoV was positively associated with the average temperature and humidity and the presence of EV and PIV4. PIV3 was positively associated with the average humidity and the presence of RSV and FluA, but negatively with the average temperature and wind velocity, and the presence of EV, HRV, and hBoV. PIV4 was positively associated with the average temperature and the presence of hCoV and RSV, however, negatively with the wind velocity. hBoV was positively associated with RSV and FluA, but negatively with the average temperature and humidity and the presence of EV and PIV3. RSV was positively associated with the presence of PIV3-4, hBoV, and FluA, but negatively with the average temperature and wind velocity. FluA was positively associated with the presence of PIV3-4, hBoV, and RSV, but negatively with the average temperature.

**Table 5:** **Multivariate logistic regression analysis of virus-meteorological and virus-virus associations**

**Covariate**	**EV**		**HRV**		**hCoV**		**PIV3**		**PIV4**		**hBoV**		**RSV**		**FluA**	
	**n (%)**	**aOR (95% CI)**	**n (%)**	**aOR (95% CI)**	**n (%)**	**aOR (95% CI)**	**n (%)**	**aOR (95% CI)**	**n (%)**	**aOR (95% CI)**	**n (%)**	**aOR (95% CI)**	**n (%)**	**aOR (95% CI)**	**n (%)**	**aOR (95% CI)**
**Temp. (°C )**	25.8 ± 3.6	**2.4 (2.1-2.7)**	21.4 ± 5.4	1.0 (0.9-1.1)	22.7 ± 4.5	**1.1 (1.0-1.2)**	14.5 ± 3.0	**0.3 (0.2-0.3)**	22.5 ± 4.2	**1.1 (1.0-1.3)**	16.3 ± 2.2	**0.7 (0.6-0.8)**	16.3 ± 4.8	**0.5 (0.5-0.6)**	16.6 ± 5.1	**0.6 (0.5-0.7)**
**Humidity (%)**	76.9 ± 9.5	**1.3 (1.1-1.6)**	72.0 ± 10.7	1.1 (1.0-1.2)	74.7 ± 11.7	**1.3 (1.1-1.5)**	70.6 ± 7.5	**1.8 (1.5-2.2)**	73.9 ± 11.1	1.1 (0.9-1.3)	63.4 ± 9.9	**0.7 (0.6-0.9)**	69.6 ± 9.8	1.2 (1.0-1.5)	69.1 ± 8.3	1.1 (0.9-1.4)
**Wind (Km/h)**	10.6 ± 3.6	1.0 (0.9-1.2)	11.4 ± 4.1	1.1 (1.0-1.2)	11.2 ± 4.6	1.0 (0.9-1.2)	10.4 ± 3.3	**0.7 (0.6-0.8)**	10.0 ± 4.0	**0.8 (0.7-0.9)**	12.4 ± 3.2	1.1 (0.9-1.3)	10.5 ± 3.4	**0.8 (0.6-0.9)**	11.3 ± 3.8	0.9 (0.8-1.1)
EV*	NA		96 (25.7)	**0.5 (0.4-0.7)**	87 (23.3)	**2.1 (1.4-3.1)**	8 (2.1)	**0.1 (0-0.2)**	68 (18.2)	1.5 (0.9-2.4)	1 (0.3)	**0 (0–0.2)**	10 (2.7)	0.6 (0.2-1.3)	8 (2.1)	0.5 (0.2-1.2)
HRV*	96 (27.2)	**0.5 (0.4-0.7)**	NA		38 (10.8)	0.8 (0.5-1.2)	30 (8.5)	**0.4 (0.3-0.7)**	31 (8.8)	0.8 (0.5-1.3)	23 (6.5)	0.7 (0.4-1.2)	17 (4.8)	0.9 (0.5-1.6)	10 (2.8)	0.5 (0.3-1.1)
hCoV*	87 (56.1)	**2.1 (1.3-3.1)**	38 (24.5)	0.8 (0.5-1.2)	NA		14 (9.0)	0.6 (0.2-1.3)	76 (49.0)	**13.1 (8.5-20.3)**	4 (2.6)	0.4 (0.1-1.4)	16 (10.3)	2.1 (1.0-4.7)	10 (6.5)	1.2 (0.5-2.8)
PIV3*	8 (5.5)	**0.1 (0–0.2)**	30 (20.7)	**0.4 (0.3-0.7)**	14 (9.7)	0.5 (0.3-1.2)	NA		17 (11.7)	0.8 (0.4-1.7)	10 (6.9)	**0.2 (0.1-0.6)**	46 (31.7)	**14.5 (7.8-27.0)**	32 (22.1)	**6.6 (3.4-12.8)**
PIV4*	68 (51.5)	**1.6 (1.0-2.6)**	31 (23.5)	0.8 (0.5-1.2)	76 (57.6)	**13.2 (8.6-20.4)**	17 (12.9)	0.9 (0.4-1.9)	NA		6 (4.5)	0.9 (0.3-2.5)	19 (14.4)	**3.1 (1.4-6.6)**	14 (10.6)	**2.7 (1.2-6.0)**
hBoV*	1 (1.3)	**0 (0–0.2)**	23 (29.5)	0.7 (0.4-1.2)	4 (5.1)	0.4 (0.1-1.2)	10 (12.8)	**0.2 (0.1-0.5)**	6 (7.7)	0.9 (0.3-2.2)	NA		15 (19.2)	**5.1 (2.4-11.0)**	11 (14.1)	**3.5 (1.6-8.0)**
RSV*	10 (13.3)	0.8 (0.3-1.8)	17 (22.7)	0.9 (0.5-1.7)	16 (21.3)	1.9 (0.8-4.7)	46 (61.3)	**13.6 (7.4-25.0)**	19 (25.3)	**2.6 (1.1-6.0)**	15 (20.0)	**4.1 (1.9-8.8)**	NA		19 (25.3)	**2.5 (1.2-5.1)**
FluA*	8 (13.8)	0.6 (0.2-1.6)	10 (17.2)	0.5 (0.3-1.1)	10 (17.2)	0.8 (0.3-2.2)	32 (55.2)	**6.6 (3.5-12.5)**	14 (24.1)	2.3 (0.9-5.6)	11 (19.0)	**3.1 (1.4-7.0)**	19 (32.8)	**2.8 (1.4-5.6)**	NA	

Categorical data are presented as number (%), continuous data are presented as mean ± SD. Viruses with no significant association are not shown.

*Adjusted for age, and all variables with *p* < 0.05 are shown in bold.

*Abbreviations:* aOR, adjusted odds ratio, 95% CI, 95% confidence interval; NA, not applicable; EV, enterovirus; HRV, human rhinovirus; hCoV, human coronavirus; PIV3-4, parainfluenza 3–4; hBoV, human bocavirus; RSV, respiratory syncytial virus; FluA, influenza A.

## Discussion

This is the first prospective study reporting the associations between meteorological parameters and co-circulation patterns of 14 common respiratory viruses. The viral detection rate among pediatric outpatients with ARIs in this study (82.3%, 884/1,074) was higher than those reported from Nanjing, China (16 viruses, 50.6%, 248/490) [19] and other countries, including Honduras (16 viruses, 75.4%, 260/345) [20] and Greece (17 viruses, 70.0%, 428/611) [6] in the same study period. Enteroviruses (EV, 34.8% and HRV, 32.9%) were most frequently detected in our outpatient children. Influenza viruses and RSV, the leading pathogens in pediatric outpatients in similar studies [6,21-23], were detected in 10.4% and 7.0% of our cases, with hCoVs (229E, OC43, HKU1, and NL63) in 14.4%, and relatively recently discovered viral pathogens hBoV and hMPV in 7.3% and 0.3% of cases, respectively (Table 3).

The viral co-detection rate (38.6%, 415/1,074) was also high among our study population. Reported rates of co-detection vary widely, from 6.1% among pediatric patients with influenza-like illness [19] to 62% among infants with acute bronchiolitis [24]. Detection of dual viruses is common, and co-detection of five [25] or even six viruses [26] is not anecdotal. All the cases with 5–7 viruses in this study were in May, the end of the cold season in the Chaoshan region. This may be in part due to past viral infections, as some viruses can still be detectable by PCR several weeks after infection [15,16]. Most studies have shown that RSV is the predominant respiratory pathogen co-detected in hospitalized children, followed by HRV, PIV, hMPV, hBoV, and FluA [25,27]. In this study, EV, HRV, hCoV, and PIV 3–4 were involved in the majority of co-detections, with EV-HRV as the most frequently co-detected pair (23% of co-detections). EV and HRV were included in the panels in many studies globally [6,7,10,11,24,28-34], and the EV-HRV pair was the most commonly detected pair among outpatient children with ARIs in Finland (19.6% of co-detections) [28] and infants with acute bronchiolitis in Brazil [10]. The co-detection rate of EV-HRV in this study is similar to that in Finland [28].

Varying detection rates of multiple viruses in different studies may reflect the differences in the study period and location, study population, environmental factors, the number of respiratory pathogens tested, and/or the diagnostic methods/techniques used. Likely reasons behind high detection rates of single and multiple viruses in this study could be due to improved recovery of viruses by using flocked swabs [35] and/or combined nasal and throat swabs [16].

There are advantages and disadvantages of multiplex PCR technique in diagnosing respiratory viral infections. While its high sensitivity and specificity facilitate simultaneous detection of a large spectrum of viruses, including those difficult to be identified by traditional methods [32], its capacity to detect low amounts of viral nucleic acids in some cases during viral incubation period, asymptomatic infection, or post-infectious shedding makes it difficult to interpret the results [30,32]. The development and validation of standardized quantitative PCR with clinically relevant cutoff values [30] or combining qPCR with serology could be helpful for etiologic understanding of simultaneous presence of multiple viruses.

Certain host-specific risk factors may predispose a child to respiratory co-infection. Younger age [11,13,14], male gender, and history of immunosuppression are associated with increased risk of viral co-detections [14]. Nonetheless, similar associations were not found in this study.

### Meteorological factors vs. viral detections

Viral co-detection is not random; clear associations for certain viral co-occurrence have been described [36]. The viruses circulating at the same time of a year are more likely to accompany each other [7,11,13]. This may be driven by meteorological factors which actually work behind seasonal variations, or by interactions of certain coexisting viruses. Temperature, humidity, and wind velocity are the most commonly studied factors significantly associated with the overall number of ARI hospitalizations and the prevalence of various respiratory viruses [37-39].

#### Temperature

The average temperature is the key climatic parameter associated with the prevalence of many respiratory viruses. Some viruses survive and/or replicate better at low temperatures, having peak prevalence in the colder months. In our study, the detection rates of PIV3, RSV, FluA, and ADV were negatively associated with temperature and were highest at temperatures between 5°C and 15°C (Tables 4 and 5), supporting the notion that low temperature is suitable for the survival of lipid-enveloped air-borne viruses [40]. Low temperatures have been found to favor RSV in southeast China [38], Malaysia [41], Nepal [23], Brazil [42] and Germany [37], influenza in Japan [43] and Germany, and ADV in Germany [37]; however, high temperatures favored PIV3 in southeast China [38,44] and Nepal [23], RSV in Singapore [45], Hong Kong [46], and Indonesia [47], and ADV in southeast China [38]. No association between temperature and FluA activity was found in Nepal [23] and Brazil [42]. In our study, other viruses such as EV, hCoV, and PIV4 were more often detected during months with higher temperatures, having peaks at temperatures between 15°C and 35°C (Tables 4 and 5). In contrast to our findings, hCoV was negatively associated with temperature, and no association between EV and temperature was found among children with ARIs in Germany [37].

#### Humidity

Association of humidity and viral detection rates has been reported from Germany [37], Singapore, Hong Kong, Brisbane, and Vancouver [40]. In this study, three viruses (EV, hCoV, and PIV3) were positively associated with the average humidity (Table 5). The optimal average humidity ranges for EV and hCoV were 70-99% and 80-99% respectively, supporting a previous finding that high average humidity (80%) had a protective effect on the survival of hCoV [48]. Our findings on PIV3 are inconsistent with animal and laboratory observations that lipid-enveloped viruses such as PIV survived better in cooler, less humid environment [49]. hBoV was negatively associated with the average humidity, and its optimal humidity was 30-69% (Tables 4 and 5). No climatic data is available for comparison regarding this virus. Previously reported association between FluA and the average humidity [40] was not found in our study.

#### Wind velocity

PIV1 and PIV3 have been reported to be negatively associated with wind velocity [37]. In low wind speed environment, viruses can easily colonize in the epithelium of upper respiratory tract [38]. An increased wind velocity is correlated with RSV activity in Germany [37]. In our study, PIV3, PIV4, and RSV were inversely associated with the wind velocity. Although we observed higher rates of EV and FluB but lower rate of HRV in low wind velocity, we could not confirm these associations by logistic regression analysis.

The underlying reasons for the observed associations between virus circulations and meteorological factors are unclear. Climate could have a direct or indirect effect on viral survival, transmission efficiency, host immunity, and social behavior change [23,37]. Cold and dry conditions might favor the transmission of viruses, and cold or rainy days could decrease outdoor activities of children and increase the probabilities of close contact and transmission of infections [37]. Holidays (supported by our data with less cases in February as Chinese new year and July-August summer holiday, Figure 2 and Additional file 3), could also play a role in an annual epidemic cycle [50]. It is likely that several factors interact in complex ways in the development of observed epidemics under optimal climatic conditions and that the contributions of individual factors vary for different viruses. Further investigations such as time series model over many years are needed to account for their inherent autocorrelations [29], and thus the observed associations between meteorological parameters and viruses in this exploratory analysis should be interpreted with caution.

### Virus-virus association

Viral co-detection patterns may be the reflection of interactions between viruses. Co-detection of viruses has been frequently reported [6,7,11,24,30,32]. Here, we have assured their associations by mathematical models (Table 5). We identified many pairs of viruses with positive associations, including EV-hCoV, hCoV-PIV4, PIV3-RSV, PIV3-FluA, PIV4-RSV, hBoV-RSV, hBoV-FluA, and RSV-FluA. Negative associations for EV-HRV, EV-PIV3, EV-hBoV, HRV-PIV3, and PIV3-hBoV were also found in this study.

### Cross-reactivity of HRV primers with EV

Both belonging to the enteroviruses genus, HRV and EV have similarities in the highly conserved sequence of the 5’ noncoding region, which is the preferred site for molecular assay development [16,17,51,52]. Cross-reactivity between the primers of HRV with EVs has been reported and is among others attributable to EV-D68, an emerging pathogen frequently undetected and misdiagnosed as HRV [17,18,52,53]. Confirmation of cross-reacting EV types in this geographic region should be done in future studies.

### Clinical significance of viral positivity

There is no consensus in the literature on the clinical implications of the viral detection and co-detection. Some studies linked multiple viral detections with fever [32], or increased hospitalization and intensive care admission [6], while others described a very similar prognosis as in single infection [10,24,25], or even milder presentations [54]. In this study, the virus-negative patients had fever more often, which may be caused by other pathogens such as bacteria. We also found that rhinorrhea was more frequently present in patients with multiple viruses than in those with a single virus, and some viruses were more (or less) likely to exist in certain age groups or were accompanied with certain symptoms. Since we did not follow the cases, the associated clinical course and outcome (such as hospitalization) remain unknown. A better understanding on the clinical courses of single and multiple viral etiologies requires further studies.

The current study has several limitations. The majority of outpatients enrolled in this study were mild and moderate cases. Therefore, we could have missed pathogens responsible for severe ARIs. As healthy or asymptomatic controls were not included, their viral carriage burdens and the actual role of virus infections could not be elucidated. Following up the cases for clinical burdens and serologic testing would be required in future studies. Air quality indicators such as Ozone and PM2.5, which might influence the host’s susceptibility or virus circulation, should be included to investigate meteorological factors.

## Conclusions

In summary, this study reports a high viral carriage in pediatric ARI cases with high viral co-detection rates mainly due to EV and HRV. There were overlapping seasonal trends of many viruses throughout the year. Meteorological factors, including temperature, humidity, and wind velocity, were associated with the viral detection rates. Statistically significant associations were present among the viruses. Further studies are needed to address polyviral etiology and viral interaction in multiple virus positive cases.

## Additional files

Additional file 1:
**Primers and probes of 14 viruses.**
Additional file 2:
**The distribution pattern of viruses in pediatric outpatients with acute respiratory infections, ARIs (n=415).**
Additional file 3:
**The number of viral co-positive cases by month.**

## Abbreviations

ARIs: Acute respiratory infections
FluA: Influenza A
FluB: Influenza B
RSV: Respiratory syncytial virus
hCoV: Human coronavirus
hMPV: Human metapneumovirus
PIV: Parainfluenza virus
HRV: Human rhinovirus
EV: Enteroviruses
ADV: Adenoviruses
hBoV: Human bocavirus
hPeV: Human parechoviruses
