# Supplementary material for: Viral aetiology of acute respiratory infections among children and associated meteorological factors in southern China
Source: BMC Infect Dis. 2015 Mar 13;15:124. doi: 10.1186/s12879-015-0863-6 (PMC4365542; doi:10.1186/s12879-015-0863-6)
Supplement: Additional file 1: — Primers and probes of 14 viruses. [file 12879_2015_863_MOESM1_ESM.docx]

**Additional File 1** Primers and probes of 14 viruses

| **Virus primer/probe** | **Sequence (5'–>3')** |
| --- | --- |
| FluA - F primer | GACAAGACCAATCCTGTCACYTCTG |
| FluA - R primer | AAGCGTCTACGCTGCAGTCC |
| FluA - probe | LCRED610-TTCACGCTCACCGTGCCCAGTGAGC-BBQ |
| FluB - F primer | TCGCTGTTTGGAGACACAAT |
| FluB - R primer | TTCTTTCCCACCGAACCA |
| FluB - probe | CYAN500-AGAAGATGGAGAAGGCAAAGCAGAACT-DB |
| ADV - F primer | CAGGACGCCTCGGRGTAYCTSAG |
| ADV - R primer | GGAGCCACVGTGGGRTT |
| ADV - probe | LCRED670-CGGGTCTGGTGCAGTTTGCCCGC-BBQ |
| EV - F primer | GGCCCTGAATGCGGCTAAT |
| EV - R primer | GGGATTGTCACCATAAGCAGCC |
| EV - probe | 6-FAM-GCGGAACCGACTACTTTGGGT-MGB-NFQ |
| RSV - F primer | ATGAACAGTTTAACATTACCAAGT |
| RSV - R primer | GTTTTGCCATAGCATGACAC |
| RSVA - probe | LCRED610-TGACTTCAAAAACAGATGTAAGCAGCTCC-BBQ |
| RSVB - probe | LCRED610-TTATGACATCAAAAACAGACATAAGCAGCTCAG-BBQ |
| hMPV - F primer | AGCTTCAGTCAATTCAACAGAAG |
| hMPV - R primer | CCTGCAGATGTYGGCATGT |
| hMPV - probe | LCRED670-TGTTGTGCGGCAGTTTTCAGACAATGC-BBQ |
| HRV-v1 - F primer* | AGSCTGCGTGGCKGCC |
| HRV-v1 - R primer* | ACACGGACACCCAAAGTAGT |
| HRV-v1 – probe* | CYAN500-TCCTCCGGCCCCTGAATGYGGCTAAYC-DB |
| HRV-v2 - F primer* | CAAGCACTTCTGTTTCCCC |
| HRV-v2 - R primer* | GGCAGCCACGCAGGC |
| HRV-v2 - probe1* | FAM-TAGACCTGGCAGATGAGGCT-BQ1 |
| HRV-v2 - probe2* | FAM-TAGTTTGGTCGATGAGGCT-BQ1 |
| HRV-v2 - probe3* | FAM-CTAGTYTGGTCGATGAGGC-BQ1 |
| PIV1 - F primer | ATCTCATTATTACCYGGACCAAGTCTACT |
| PIV1- R primer | CATCCTTGAGTGATTAAGTTTGATGAATA |
| PIV1 - probe | CYAN500-AGGATGTGTTAGAYTACCTTCATTATCAATTGGTGATG-DB |
| PIV2 - F primer | CTGCAGCTATGAGTAATC |
| PIV2 - R primer | TGATCGAGCATCTGGAAT |
| PIV2 - probe | LCRED610-AGCCATGCATTCACCAGAAGCCAGC-BBQ |
| PIV3 - F primer | ACTCTATCYACTCTCAGACC |
| PIV3 - R primer | TGGGATCTCTGAGGATAC |
| PIV3 - probe | LCRED670-AAGGGACCACGCGCTCCTTTCATC-BBQ |
| PIV4 - F primer | GATCCACAGCAAAGATTCAC |
| PIV4 - R primer | GCCTGTAAGGAAAGCAGAGA |
| PIV4 - probe | HEX-TATCATCATCTGCCAAATCGGCAA-BHQ1 |
| hCoV1 - F primer | GGTGGYTGGGAYGATATGTTACG |
| hCoV1 - R primer | KRTTTGGCATAGCACGATCACA |
| hCoV1 - probe | 6-FAM-ATGTTGACAAYCCTGTWCTTATGGGTTGGG-MGB-NFQ |
| hCoV2 - F primer | GCTRAGCATGATTTCTTTACTTGG |
| hCoV2 - R primer | CARTYTTKTTCATCAAAGTTACGCA |
| hCoV2 - probe | 6-FAM-CAGARTCATTTATGGTAATGTTAGTAGACA-MGB-NFQ |
| hBoV - F primer | CAAATCTCTTCTGGCTACACG |
| hBoV - R primer | CTCTGCGATCTCTATATTGAAGG |
| hBoV - probe | LCRED670-ATGTTGCCGCCAGTAACTCCACC-BBQ |
| hPeV - F primer | CTGGGGCCAAAAGCCA |
| hPeV - R primer | GGTACCTTCTGGGCATCCTTC |
| hPeV - probe | LCRED610-AAACACTAGTTGTAWGGCCC--BBQ |
| EAV - F primer | CATCTCTTGCTTTGCTCCTTAG |
| EAV - R primer | AGCCGCACCTTCACATTG |
| EAV- probe | 6-FAM-CGCGCTCGCTGTCAGAACAACATTATTGCCCACAGCGCG-NFQ |

*HRV-v2 (HRV version 2) was used later to confirm the results of HRV-v1 (HRV-version 1) and EV co-positive cases.

Abbreviations: FluA, influenza A; FluB, influenza B; ADV, adenovirus; EV, enterovirus; RSV, respiratory syncytial virus; hMPV, human metapneumovirus; HRV, human rhinovirus; PIV1-4, parainfluenza 1-4; hCoV, human coronavirus; hBoV, human bocavirus; hPeV, human parechoviruses; EAV, equine arteritis virus
