# Supplementary material for: Viral aetiology of acute respiratory infections among children and associated meteorological factors in southern China
Source: BMC Infect Dis. 2015 Mar 13;15:124. doi: 10.1186/s12879-015-0863-6 (PMC4365542; doi:10.1186/s12879-015-0863-6)
Supplement: Additional file 2: — The distribution pattern of viruses in pediatric outpatients with acute respiratory infections, ARIs (n=415). [file 12879_2015_863_MOESM2_ESM.docx]

**Additional File 2** The distribution pattern of viruses in pediatric outpatients with acute respiratory infections, ARIs (n=415)

| **No. of viruses** | **Co-detection pattern** | **No. of cases** |
| --- | --- | --- |
| 2 viruses | EV+HRV | 72 |
|  | EV+hCoV | 31 |
|  | EV+PIV4 | 17 |
|  | EV+PIV1 | 5 |
|  | EV+PIV2 | 4 |
|  | EV+FluB | 2 |
|  | EV+PIV3 | 1 |
|  | HRV+PIV3 | 13 |
|  | HRV+hBoV | 12 |
|  | HRV+FluB | 12 |
|  | HRV+PIV4 | 5 |
|  | HRV+hCoV | 3 |
|  | HRV+FluA | 2 |
|  | HRV+RSV | 2 |
|  | HRV+PIV2 | 1 |
|  | PIV3+RSV | 18 |
|  | PIV3+FluA | 13 |
|  | PIV3+PIV4 | 4 |
|  | PIV3+PIV1 | 2 |
|  | PIV3+hBoV | 2 |
|  | PIV3+ADV | 1 |
|  | PIV3+hCoV | 1 |
|  | PIV3+PIV2 | 1 |
|  | PIV4+hCoV | 15 |
|  | PIV4+hBoV | 2 |
|  | PIV4+FluA | 1 |
|  | hBoV+FluA | 5 |
|  | hBoV+RSV | 5 |
|  | hBoV+ADV | 2 |
|  | hBoV+PIV2 | 1 |
|  | hBoV+hMPV | 1 |
|  | hBoV+PIV1 | 1 |
|  | FluB+PIV1 | 4 |
|  | RSV+FluA | 1 |
|  | RSV+ADV | 1 |
|  | ADV+PIV2 | 1 |
|  | PIV2+hCoV | 1 |
| 3 viruses | PIV4+hCoV+EV | 28 |
|  | PIV4+hCoV+HRV | 8 |
|  | PIV4+hCoV+FluB | 1 |
|  | PIV4+hCoV+hBoV | 1 |
|  | PIV4+hBoV+FluA | 1 |
|  | PIV4+hBoV+RSV | 1 |
|  | EV+HRV+hCoV | 5 |
|  | EV+HRV+PIV4 | 5 |
|  | EV+HRV+PIV2 | 3 |
|  | EV+HRV+FluB | 2 |
|  | PIV3+RSV+HRV | 4 |
|  | PIV3+RSV+hBoV | 3 |
|  | PIV3+RSV+FluA | 2 |
|  | PIV3+RSV+hCoV | 2 |
|  | PIV3+RSV+ADV | 1 |
|  | PIV3+RSV+EV | 1 |
|  | PIV3+FluA+HRV | 4 |
|  | PIV3+FluA+ADV | 1 |
|  | PIV3+FluA+EV | 1 |
|  | PIV3+FluA+PIV2 | 1 |
|  | PIV3+FluA+PIV4 | 1 |
|  | PIV3+PIV4+ADV | 1 |
|  | PIV3+PIV4+PIV1 | 1 |
|  | PIV3+PIV4+RSV | 1 |
|  | PIV3+hBoV+EV | 1 |
|  | PIV3+ADV+EV | 1 |
|  | HRV+hCoV+PIV2 | 5 |
|  | HRV+hCoV+PIV3 | 3 |
|  | HRV+hBoV+hCoV | 2 |
|  | HRV+hBoV+FluA | 1 |
|  | HRV+hBoV+ADV | 1 |
|  | HRV+hBoV+RSV | 1 |
|  | HRV+hBoV+PIV2 | 1 |
|  | HRV+hBoV+PIV4 | 1 |
|  | HRV+RSV+PIV4 | 1 |
|  | FluB+PIV1+HRV | 3 |
|  | FluB+hBoV+hMPV | 1 |
|  | FluB+PIV4+EV | 1 |
|  | hCoV+PIV2+EV | 3 |
|  | hCoV+FluB+EV | 2 |
|  | hCoV+PIV2+PIV3 | 1 |
|  | PIV1+hCoV+hBoV | 1 |
|  | ADV+RSV+HRV | 1 |
|  | ADV+PIV3+PIV2 | 1 |
| 4 viruses | EV+HRV+PIV4+hCoV | 6 |
|  | EV+HRV+PIV2+hCoV | 2 |
|  | EV+PIV1+PIV4+hCoV | 1 |
|  | EV+PIV2+PIV4+hCoV | 1 |
|  | HRV+PIV4+FluB+hCoV | 2 |
|  | HRV+RSV+PIV3+ADV | 1 |
|  | HRV+PIV2+PIV3+hBoV | 1 |
|  | FluA+ADV+PIV3+hBoV | 1 |
|  | FluA+RSV+hBoV+FluB | 1 |
|  | FluA+RSV+HRV+hBoV | 1 |
|  | FluA+RSV+HRV+PIV3 | 1 |
|  | FluA+ADV+PIV2+PIV3 | 1 |
|  | FluA+RSV+PIV3+hBoV | 1 |
|  | FluA+RSV+PIV3+hCoV | 1 |
|  | FluA+RSV+PIV4+hCoV | 1 |
|  | FluA+RSV+PIV3+PIV4 | 1 |
|  | RSV+PIV3+PIV4+hCoV | 1 |
|  | RSV+HRV+PIV1+hBoV | 1 |
|  | RSV+HRV+PIV3+hBoV | 1 |
|  | RSV+HRV+PIV3+PIV4 | 1 |
| 5 viruses | RSV+PIV4+hCoV+FluA+EV | 2 |
|  | RSV+PIV4+PIV3+hCoV+EV | 1 |
|  | RSV+PIV4+PIV3+FluA+EV | 1 |
|  | RSV+PIV4+hCoV+FluA+PIV3 | 1 |
| 6 viruses | EV+RSV+PIV4+hCoV+FluA+PIV2 | 3 |
|  | EV+HRV+RSV+PIV4+hCoV+FluA | 1 |
|  | RSV+HRV+PIV2+PIV3+PIV4+hCoV | 1 |
|  | FluA+RSV+PIV2+PIV3+PIV4+hCoV | 1 |
| 7 viruses | EV+RSV+PIV4+hCoV+FluB+PIV2+PIV3 | 1 |

Abbreviations: EV, enterovirus; HRV, human rhinovirus; hCoV, human coronavirus; PIV1-4, parainfluenza 1-4; hBoV, human bocavirus; RSV, respiratory syncytial virus; FluA, influenza A; FluB, influenza B; ADV, adenovirus; hMPV, human metapneumovirus
